# Raman spectroscopy biochemical characterisation of bladder cancer cisplatin resistance regulated by FDFT1: a review

**DOI:** 10.1186/s11658-022-00307-x

**Published:** 2022-01-29

**Authors:** M. Kanmalar, Siti Fairus Abdul Sani, Nur Izzahtul Nabilla B. Kamri, Nur Akmarina B. M. Said, Amirah Hajirah B. A. Jamil, S. Kuppusamy, K. S. Mun, D. A. Bradley

**Affiliations:** 1 Department of Physics, Faculty of Science, University of Malaya, 50603 Kuala Lumpur, Malaysia; 2 Department of Pharmaceutical Life Sciences, Faculty of Pharmacy, University of Malaya, 50603 Kuala Lumpur, Malaysia; 3 Department of Surgery, University of Malaya, 50603 Kuala Lumpur, Malaysia; 4 Department of Pathology, Faculty of Medicine, University of Malaya, 50603 Kuala Lumpur, Malaysia; 5 Centre for Applied Physics and Radiation Technologies, Sunway University, Jalan University, 46150 Petaling Jaya, Malaysia; 6 Department of Physics, University of Surrey, Guildford, GU2 7XH UK

**Keywords:** Bladder cancer, Diagnostic, FDFT1, Cisplatin chemoresistance, Raman spectroscopy

## Abstract

Bladder cancer is the fourth most common malignancy in males. It can present across the whole continuum of severity, from mild through well-differentiated disease to extremely malignant tumours with poor survival rates. As with other vital organ malignancies, proper clinical management involves accurate diagnosis and staging. Chemotherapy consisting of a cisplatin-based regimen is the mainstay in the management of muscle-invasive bladder cancers. Control via cisplatin-based chemotherapy is threatened by the development of chemoresistance. Intracellular cholesterol biosynthesis in bladder cancer cells is considered a contributory factor in determining the chemotherapy response. Farnesyl-diphosphate farnesyltransferase 1 (FDFT1), one of the main regulatory components in cholesterol biosynthesis, may play a role in determining sensitivity towards chemotherapy compounds in bladder cancer. FDFT1-associated molecular identification might serve as an alternative or appendage strategy for early prediction of potentially chemoresistant muscle-invasive bladder cancer tissues. This can be accomplished using Raman spectroscopy. Developments in the instrumentation have led to it becoming one of the most convenient forms of analysis, and there is a highly realistic chance that it will become an effective tool in the pathology lab. Chemosensitive bladder cancer tissues tend to have a higher lipid content, more protein genes and more cholesterol metabolites. These are believed to be associated with resistance towards bladder cancer chemotherapy. Herein, Raman peak assignments have been tabulated as an aid to indicating metabolic changes in bladder cancer tissues that are potentially correlated with FDFT1 expression.

**Supplementary Information:**

The online version contains supplementary material available at 10.1186/s11658-022-00307-x.

## Introduction

Bladder cancer is one of the most prevalent urogenital malignancies [1], accounting for some 5 to 10% of total male malignancies worldwide, with a male-to-female ratio varying from 2: 1 to 6:1 in various regions [2]. Based on transurethral resections of bladder tumours (TURBT), approximately 70–80% of patients are diagnosed with non-muscle invasive bladder cancer (NMIBC), with a recurrence and progression rate to muscle invasive bladder cancer (MIBC) of 50–70% [3]. The incidence of MIBC at first presentation is about 20%. MIBC requires a more radical treatment approach that can contribute to increased morbidity and poorer overall survival. Treatment for MIBC generally involves radical cystectomy (RC) with urinary diversion, representing the standard of care [4] with almost 50% of cases progressing to metastatic state within 2 to 3 years [5].

However, new data show improved overall survival with the use of cisplatin (cis-diamminedichloroplatinum [II])-based chemotherapy in a neoadjuvant setting followed by RC [6]. Bladder sparing methods are also advocated, using a combination of cisplatin-based chemotherapy and radiotherapy to avoid the morbidity associated with RC [7].

Cisplatin-based chemotherapy improves survival but may also be related to resistance and toxicity that may hinder capability to undergo RC or achieve benefit with radiotherapy. Resistance to cisplatin-based chemotherapy may mean it is not an option for patients who progress to metastatic state after radical treatment. There are currently no well-defined prognostic markers that can identify patients that are at high risk of developing resistance. A comprehensive understanding of patient subsets predicted to develop resistance towards cisplatin is essential to develop new therapeutic modalities.

Cholesterol is essential for cellular signals, including proliferation, and it is synthesised by virtually all tissues in the human body. The liver, intestine, adrenal cortex, and reproductive tissues give the largest contributions to the body’s cholesterol pool. An imbalance in cholesterol regulation can lead to an elevation in circulating levels of plasma cholesterol, with the potential for the development of many cancers [8]. Cholesterol biosynthesis involves the mevalonate pathway. The upstream HMG-CoA pathway targeted by the drug simvastatin has shown to be a promising target to increase sensitivity towards doxorubicin in an in-vitro bladder cancer model [9]. Statin-induced reduction in intracellular cholesterol levels also has been shown to be correlated with inhibiting cancer cell line growth [10]. However, studies of the effect of cholesterol and the use of cholesterol-lowering approaches targeting HMG-CoA have given inconclusive results [11]. There is a need to elucidate the linchpin molecule that governs this process.

Chemoresistance and tumour progression may involve farnesyl-diphosphate farnesyltransferase 1 (FDFT1), a gene that encodes the membrane-associated enzyme squalene synthase, which is the first specific enzyme in cholesterol biosynthesis [12, 13]. However, its role in tumour progression is cancer-specific: FDFT1 has been implicated as a potential oncogene and as a tumour-suppressive gene in different types of cancer [14, 15]. In liver, lung, prostate, breast, ovary, bladder, cervix, thyroid, and esophageal cancers, FDFT1 is highly expressed, while in colorectal, colon, testicular, uterine, pancreas, and kidney tumours, its expression is downregulated [14–16]. In bladder cancer specifically, FDFT1 has been suggested as a predictive marker for drug sensitivity to chemotherapy compounds [17].

Other than the standard biopsy-staining approach, several groups have researched alternative techniques that may improve and/or enhance the diagnosis of bladder cancer [18]. These include fluorescent cystoscopy [19–21], electrical impedance spectroscopy [22], virtual cystoscopy with computed tomography (CT), and modulated resonance imaging (MRI) [23]. They can reduce time-consuming aspects of detecting tumour sites. Further to this, protein and DNA markers are used in urine analysis [24, 25], aiding in the detection of a tumour’s presence and further supporting analyses of changes in tissue arrangement and configuration. In some techniques, the absence of contrast agents means that changes in composition and morphology cannot be localised.

The specificity and accuracy offered by Raman spectroscopy allows it to be employed as a non-invasive diagnostic tool for bladder cancer, providing for observation of pathogenesis and any biomolecular heterogeneity associated with cancer progression. Raman spectroscopy can also be applied in vivo during cystoscopy or in vitro to cells obtained from urine cytology samples.

The inelastic Raman scattering process concerns photons interacting at the molecular level, resulting in a shift in wavelength [26]. Examples of prior Raman spectrographic analyses in pathology have included examining for the existence of positives with dubious fluorescence [26]. The transmitted scattered photons result in the formation of a series of peaks, each characterising a molecular bond, providing a corresponding molecular fingerprint for a specimen [26]. Accordingly, Raman spectroscopy can assist in conjunction with other diagnostic strategies, providing user-friendly, real-time analysis. Current research focuses on the development of discriminatory cholesterol profiles via Raman spectroscopy, aimed at stratifying chemosensitivity status in bladder cancer tissues using this rapid and reagent-free tool.

## Bladder carcinoma

Bladder cancer management depends on both the histotype and patients’ risk factors. It is among the most costly cancers to treat, particularly in patients with advanced cancers [27]. There have been minor improvements in survival rates over the years [28], partly in response to treatment resistance. There have been multiple efforts in terms of early detection, better therapies, and survival improvement. Understanding the biology and molecular interplay underlying tumour progression and treatment failure supports the development of tools in the management of bladder cancer [29], specifically treatment response.

### Screening and diagnosing bladder cancer and treatment evaluation

Cystoscopy (bladder endoscopy) is used in the advent of haematuria or voiding and storage symptoms, which are potential indicators of bladder cancer. Exfoliated urothelial cells from the urethra and voided urine are viewed using appropriate microscopy. When suspicious growth is identified during cystoscopy, TURBT is typically performed, providing a histopathological diagnosis.

Most bladder cancers are papillary, and some solid or mixed. Histopathological examination determines the bladder cancer subtype, grade, and tumour (T) staging, and treatment decisions can be made based on this and other information. The most common subtype is transitional cell carcinoma, making up at least 90% of all bladder cancer subtypes. Other less common subtypes include those with squamous cell or glandular differentiation. The tumour is graded based on the WHO 2004/2016 Classification of Tumours of the Urinary System and Male Genital Organs [30], as low grade (LG), high grade (HG) or papillary urothelial neoplasm of low malignant potential (PUNLMP) based on the extent of cellular differentiation and anaplastic features.

**Fig. 1:**
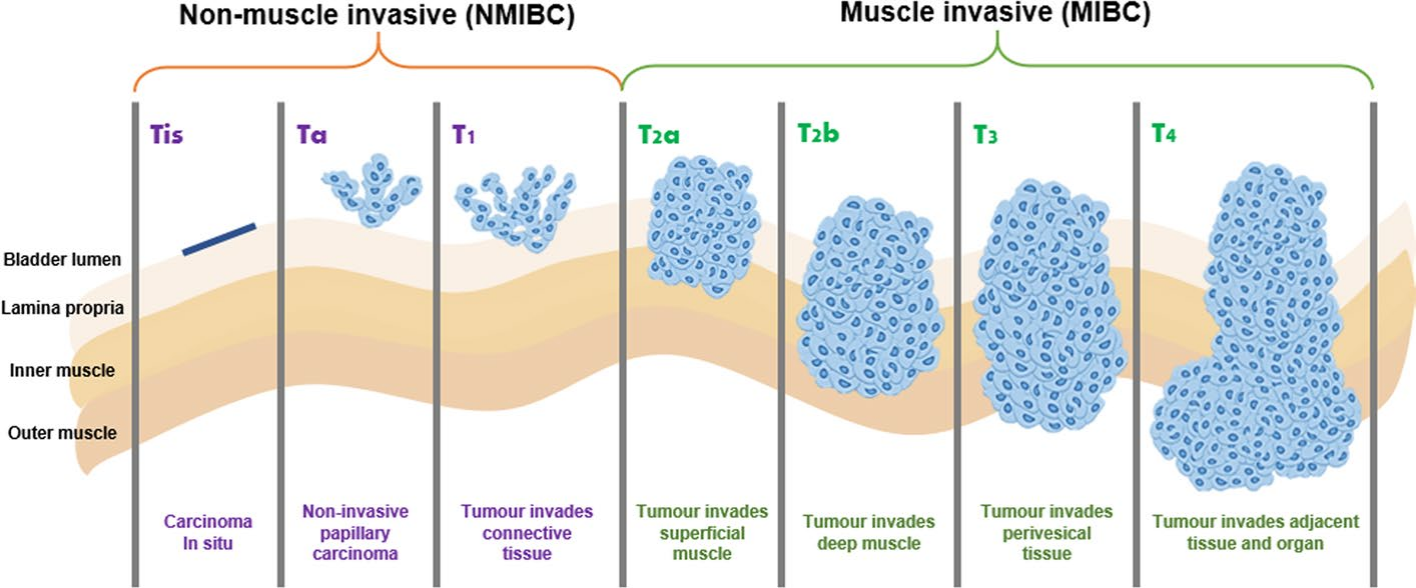
The classification of stages in diagnosing bladder cancer into non-muscle invasive bladder cancer (NMIBC) and muscle invasive bladder cancer (MIBC)

Staging of bladder cancer is based on the UICC TNM system of classification (2017), i.e., tumour (T), node (N), metastasis (M), determining the pathological and clinical stage of the disease. The pathological assessment of the tumour provides information on the depth of invasion of the tumour (T) in relation to the bladder, with evidence of infiltration of lamina propria, muscle, perivesical tissue and adjacent structures (Fig. 1). Based on the T stage of bladder cancer, the malignancy is broadly classified as non-muscle invasive (NMIBC) or muscle invasive (MIBC). Another important stage of bladder cancer is the presence of carcinoma in situ (CIS): a high-grade, non-invasive urothelial carcinoma that is deemed to be a significant lesion. This is often missed due to its flat appearance, which may mimic an inflammatory lesion. Lymph node (N) status is also obtained from pathological assessment, which may indicate infiltration from specimens available from pelvic lymph node dissection during RC. However, in patients not undergoing radical surgery, staging would then rely on radiological imaging modalities, such as a CT scan to assess the lymph node and metastasis (M) status. This information, from tumour subtype, grade, clinical and pathological TNM staging, determines the treatment option. High-grade tumours are more likely to recur and progress as seen with MIBC patients, more than half of whom are at risk of developing metastasis.

### Treatment of bladder carcinoma

Treatment of bladder cancer depends on risk stratification, which is vital information for enabling the clinician to estimate the probability of patients presenting with recurrence and the risk of progression. Factors taken into consideration include tumour staging, the presence of CIS, and the size and multiplicity of the lesions. The treatment for NMIBC is to surgically remove the lesion via TURBT, with the possibility of either supplementing the treatment with intravesical therapy using chemotherapy (mitomycin C) or intravesical immunotherapy using bacillus calmette-guerin (BCG). The use of BCG is usual in confirmed cases of HGT1 disease or when there is CIS in the resected specimen. These adjuvant intravesical therapies seek to reduce the risk of recurrence and progression to MIBC. This is followed by surveillance cystoscopy at regular intervals, looking for recurrence and progression.

When a patient is diagnosed with MIBC, with the majority HG, the standard of care is to perform radical cystectomy with urinary diversion, including pelvic lymph node dissection. Current evidence and guidelines include the role of neoadjuvant cisplatin-based chemotherapy in viable practice. Counselling of patients is supported by an improved 10-year overall survival of some 6% [31], with 16% reduction in the risk of mortality [32]. This is also a strategy to treat the possible micrometastasis present in the patient. The use of neoadjuvant chemotherapy also gives an indication as to whether the patient would respond to future adjuvant cisplatin-based chemotherapy if recurrence and progression of disease is identified in follow-up.

Adjuvant therapy is not indicated for treatment of MIBC after RC with or without neoadjuvant chemotherapy. In this regard, some patients will not accept losing the bladder and the need to cope with urinary diversion through use of a stoma, referred to as the ileal conduit. Thus, in management of lymph nodes, an alternative bladder sparing strategy could be considered, using cisplatin-based chemotherapy followed by radiotherapy. This should include local therapy, performing TURBT to ensure all of the lesion in the bladder is resected.

#### Cisplatin-based chemotherapy in bladder cancer

In bladder cancer, cisplatin is the backbone for chemotherapy regimens, either as a part of the methotrexate, vinblastine, adriamycin and cisplatin (MVAC) or gemcitabine and cisplatin (Gem-Cis) drug combinations. DNA is the main cellular target. As cisplatin diffuses into the cell, it reacts with the DNA strands. Then, the cisplatin will be activated after replacing its two chloride ligands with water molecules. Cellular death through activation of the apoptotic tract is the final consequence of cisplatin-mediated DNA damage.

However, cisplatin-based chemotherapy against bladder cancer is challenged by chemoresistance. The response rate towards this first line chemotherapy barely exceeds 50% [33] and patients who suffer recurrence after cisplatin-based chemotherapy are often resistant to second line chemotherapy, with a median progression-free survival (PFS) of 3–4 months [34]. Therefore, patients would likely benefit from a predictive test that can be performed on the biopsy sample taken during diagnosis or TURBT. A comprehensive clinical and molecular understanding of emerging pathways in chemoresistance is thus fundamentally essential in developing the predictive marker.

Bladder cancer cells have been shown to develop resistance towards cisplatin via various mechanisms, including drug inactivation, drug target alteration, drug efflux, DNA damage repair, cell death inhibition and the epithelial–mesenchymal transition (EMT) [33]. Recently, an impaired cholesterol metabolism has been shown to be one of the most important mechanisms regulating the cellular response towards chemotherapy [35].

## Role of abnormal cholesterol metabolism and FDFT1 in tumour progression and chemoresistance

Cholesterol is an essential component of the cellular membrane. Distributed between hydrocarbon chains, it plays a pivotal role during membrane assembly and in the stability, architecture, dynamics, and function of the plasma cell membrane. It also modulates transmembrane receptor signalling, is involved in vesicle trafficking, and serves as the precursor of steroid hormones and sterols during steroidogenesis [36, 37]. Thus, cholesterol moderates multiple biological responses and regulates various cellular functions, including membrane biogenesis, cell growth, proliferation, apoptosis, and migration [38, 39], and is increasingly implicated in carcinogenesis and chemoresistance [39, 41–43]. Here, we briefly review how cholesterol is synthesised, how cancer cells reprogram cholesterol metabolism to promote carcinogenesis and treatment resistance to chemotherapy [8, 39, 40], and the emerging role of FDFT1 in tumour progression and chemoresistance.

### Overview of cholesterol metabolism

**Fig. 2:**
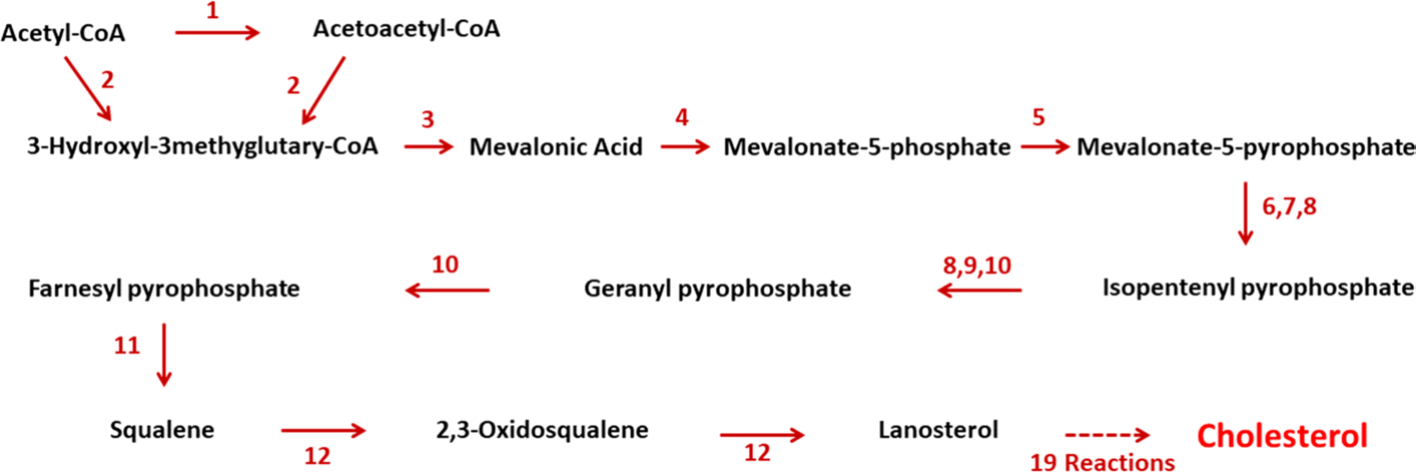
The cholesterol biosynthesis pathway. (1) Thiolases or acetyl-coenzyme A acetyltransferases, (2) hydroxy-3-methylglutaryl-CoA synthase, (3) hydroxy-3-methylglutaryl-CoA reductase, (4) mevalonate-3-kinase or me- valonate-5-kinase, (5) mevalonate-3-phosphate-5-kinase or phosphomevalonate kinase, (6) mevalonate-5-phosphate decarboxylase, (7) mevalonate pyrophosphate decarboxylase, (8) isopentenyl phosphate kinase, (9) isopentenyl pyrophosphate isomerase, (10) farnesyl-diphosphate synthase, (11) squalene synthase, or FDFT1, (12) squalene monooxygenase or squalene epoxidase. There are 19 reactions, including multiple demethylations, desaturations, isomerizations, and reductions [44]

Approximately half of the cholesterol in the body is derived from de novo biosynthesis. Every mammalian cell can synthesise cholesterol, although the liver, intestine, adrenal cortex, and reproductive tissues (including the ovaries, testes, and placenta) make the largest contributions to the body’s cholesterol pool. Biosynthesis in the liver accounts for approximately 10%, and in the intestines approximately 15% of the amount produced each day. Cholesterol is a 27-carbon and tetracyclic ring steroid catalysed by a series of more than 26 separate enzymatic reactions in several subcellular compartments [37, 44, 45]. The process of cholesterol synthesis is comprised of several major steps as depicted in Fig. 2.

Apart from de novo cholesterol biosynthesis, most cells can also acquire cholesterol from low-density lipoprotein (LDL) taken up from the circulation via LDL receptor-mediated (LDLR-mediated) endocytosis. Enterocytes absorb dietary cholesterol from the intestinal lumen via cholesterol transporters, the clathrin adaptor, and the adaptor protein [46, 47]. Within the cell, cholesterol is dynamically transported to its destination membranes, where it fulfills structural and functional needs. Cholesterol that exceeds cellular demand is either exported from the cell by ATP-binding cassette (ABC) transporters, converted to less toxic cholesteryl esters (CEs) by acyl-coenzyme cholesterol acyltransferases (ACATs) and stored in lipid droplets, or secreted within lipoproteins [48].

Cholesterol concentrations at both the cellular and systemic levels are maintained with fine-tuned regulations. The master transcriptional regulators governing cholesterol homeostasis include sterol regulatory element–binding protein-2 (SREBP-2), liver X receptors (LXRs) and nuclear factor erythroid 2 related factor-1 (NRF1) [49]. Accumulation of cholesterol and cholesterol-derived oxysterols inactivates the SREBP-2 pathway, thereby downregulating cholesterol biosynthesis and uptake. In addition, desmosterol, the immediate precursor of cholesterol and oxysterols, binds and activates LXRs, thereby enhancing the expression of genes involved in cholesterol efflux, such as the ATP-binding cassette subfamily A member 1 (ABCA1) [50]. These regulatory pathways function in a coordinated and opposing manner under conditions of cholesterol deficiency, ensuring an increase in cholesterol biosynthesis and uptake as well as a decline in cholesterol efflux and esterification.

### Reprogrammed cholesterol metabolism in cancer cells

Reprogrammed lipid metabolism is now established as a hallmark of cancer, with accelerated cholesterol metabolism increasingly associated with many types of cancer [8, 39, 40]. Cancer cells require excess cholesterol and intermediates of the cholesterol biosynthetic pathway to maintain accelerated levels of cell growth and proliferation. Cholesterol is also capable of regulating multiple signalling pathways involved in carcinogenesis, cancer cell migration, and tumour progression, but it has also recently been strongly associated with chemoresistance [39, 41–43]. Indeed, transformed cells reprogram cholesterol metabolism by increasing its uptake and de novo biosynthesis, or by deregulating its efflux.

The master transcription factor SREBP2 and its downstream targets, including mevalonate pathway enzymes, are significantly upregulated in various tumours [51]. This can be accompanied by increased levels of the enzyme SQLE, for example in advanced-stage prostate cancer, indicating a greater reliance on cholesterol synthesis [52]. Cholesterol biosynthesis also has a critical role in maintaining cancer stem cells: it activates cellular signalling pathways downstream of hedgehog, notch and receptor tyrosine kinases [53]. Increased cholesterol uptake is also observed in cancer cells. As an example, anaplastic large cell lymphoma cells fully rely on cholesterol uptake to acquire cholesterol. They actively upregulate LDLR, which takes up exogenous cholesterol as an alternative strategy to support proliferation [55].

The mevalonate pathway has also been implicated in oncogenic pathways, with gain of oncogenes also corresponding with fluctuations in cholesterol biosynthesis, while tumour suppressors antagonise this overactivated state and maintain cholesterol homeostasis. For example, activation of Akt has been shown to cause the upregulation of SREBP and its target genes in the cholesterol pathway, suggesting a positive correlation between oncogene activation and cholesterol metabolism [56]. The oncogene MYC has also been shown to be responsible for upregulation of the mevalonate pathway in patient-derived brain tumour-initiating cells [57]. The tumour suppressor p53 upregulates the cholesterol efflux transporter ABCA1, thereby restricting SREBP2 maturation and subsequently repressing the mevalonate pathway. In prostate cancer [59] and hepatocellular carcinoma [60], the loss of the tumour suppressor phosphatase and tensin homolog (PTEN) activates PI3K–Akt signaling and leads to increased cholesterol uptake, facilitating cancer progression.

Increased resistance to apoptotic signals was also reportedly linked to high mitochondrial cholesterol in a few types of cancer. Proteins such as STAR and STARD3, which are responsible for initiating the transportation of cholesterol to the mitochondria, were shown to be associated with increased cancer cell proliferation and a decreased therapeutic response to chemotherapy in breast cancer cells [61]. Numerous reports suggest that an alteration in cholesterol metabolism contributes to the ineffectiveness of various cancer therapies. An increase in the mitochondrial cholesterol level also causes chemoresistance in hepatocellular carcinoma by increasing the expression of P-glycoprotein (P-gp) through PI3K/mTOR signaling, thereby diminishing the efficacy of doxorubicin [62]. High cholesterol content hampers the sensitivity of paclitaxel and cisplatin through upregulation of the drug efflux pumps, along with an increase in cholesterol receptor (LXRɑ/β) expression [63]. A similar observation was reported in lung adenocarcinoma where pre-treatment or co-treatment of cholesterol with carboplatin decreased the cytotoxic potential of these drugs in A549 cells [64].

It is evident that deregulated cholesterol metabolism promotes tumour progression and hampers treatment strategies in various cancers. Understanding the mechanistic role of cholesterol in bladder cancer carcinogenesis and the therapeutic implications is an emerging research topic. Recent studies reported the oncogenic roles of the cholesterol metabolite 25-hydroxycholesterol in bladder cancer and promoted chemoresistance in T24 and RT4 bladder cancer cells [65]. Therefore, improved understanding on the regulation of cholesterol metabolism has important implications for exploring new therapeutic strategies for the management and treatment of bladder cancer.

### Role of FDFT1 in cancer progression and chemoresistance

FDFT1 is a gene that encodes the membrane-associated enzyme squalene synthase, which is the first specific enzyme in cholesterol biosynthesis that is increasingly implicated in tumour progression and the development of therapeutic resistance to anticancer drugs. Cholesterol biosynthesis pathways include branches producing sterol and non-sterol isoprenoids. FDFT1 has a critical regulatory role, as the specific enzyme in the sterol branch catalyses the conversion of farnesyl diphosphate (FPP) into squalene, leading to the synthesis of cholesterol. As the FPP is located at the end of the biosynthesis cycle of isoprenoid, its modification into squalene catalysed by FDFT1 action is the first stage of cholesterol biosynthesis, making it an obvious target for therapeutic intervention [66]. Of cellular and molecular importance in the mevalonate pathway is the prenylation of isoprenoids. This important post-translational modification along with activation of regulatory proteins, such as G-proteins, Ras, and p21, is essential for intracellular signalling and cell growth [15].

In prostate cancer cell lines, inhibiting FDFT1 has been shown to inhibit cell proliferation. FDFT1 transcript levels have been found to be significantly greater in prostate cancer tissues than in benign tissues, and higher in very aggressive cancers than in those that are moderately aggressive [14]. These findings in prostate cancer may be attributed to the androgen regulation of FDFT1 [16].

In contrast to these findings, FDFT1 is identified as a tumour suppressor gene in the bladder cancer model. Apart from being highly expressed in cisplatin-sensitive bladder cancer cell lines compared to its resistant counterpart, knockdown of FDFT1 in bladder cancer cells has resulted in acquisition of mesenchymal morphology and increased vimentin expression [12]. Indeed, FDFT1 has been shown to be overexpressed in superficial bladder cancer samples compared to muscle invasive bladder carcinoma [13]. This could be partly explained by the role of FDFT1 in directing the FPP substrate towards sterol and non-steroid branches. In tumours where FDFT1 is downregulated, isoprenoids can accumulate, providing more substrate for protein prenylation, causing uncontrolled cell growth.

Interestingly, FDFT1 is also among the top 10% genes overexpressed in bladder cancers that are sensitive to the anti-tumour drugs vandetanib and tipifarnib [17, 67] and in esophageal cancer cell lines that are sensitive to docetaxel, paclitaxel, and doxorubicin [68]. FDFT1 is also among the top 1% of genes with copy number gain in multiple cancer cell lines that are sensitive to panobinostat [67]. All these data suggest the role of FDFT1 in conferring sensitivity towards chemotherapy, and its potential as a predictive marker for drug sensitivity. As such, cholesterol- and FDFT1-associated biochemical characterisation might aid the prediction of chemosensitivity in bladder cancer patients. This can be achieved using Raman spectroscopy, making it a feasible complementary predictive tool.

## Raman spectroscopy

Due to the attainability of molecular fingerprints, vibrational spectroscopy has become a remarkable technique to analyze substances at the molecular level. Raman spectroscopy, as a vibrational spectroscopy, enables the determination and characterization of the chemical properties of a material. Besides identifying the presence of molecules, Raman spectroscopy can examine the intramolecular bonds and deliver quantified results by allowing the determination of the vibrational frequencies of the chemical bonds. It is undeniable that the advantageous output of Raman spectroscopy has attracted major interest in industries such as geology, mineralogy and electronic fields, but due to the accessibility of Raman spectroscopy ex vivo and in vivo it is also widely used for research in describing biomedical complications [69]. This mechanism is used extensively to diagnose disease as it can discriminate diseased tissues from healthy tissues based on biomolecular changes. Recent diagnostic studies on Alzheimer’s disease [70], bacterial infection such as typhoid and tuberculosis [71], and cardiovascular disease [72] demonstrated the promising capability of Raman spectroscopy for early detection.

### Mechanism of Raman spectroscopy

The analytical method referred to as Raman spectroscopy is based on the inelastic scattering of incident monochromatic light by molecules within specimens. Elastic scattering, referred to as Rayleigh scattering, contrasts with this. The shift in energy from incident light differentiates Raman from Rayleigh scattering, but Rayleigh scattering dominates over the former. Although only 1 in 10^7^ photons subjected to Raman inelastic scattering [73], it is nevertheless a powerful technique.

The interaction of incident light with the molecular bonds in the material leads to molecules transitioning from one vibrational energy state to another. Then, the molecules relax and de-excite to a final vibrational energy state via radiative transitions, emitting scattered photons. The energy of the scattered photon is the difference between the energy of the initial and final vibrational states, with the energy of the scattered photons being unique to specific molecular bonds. The loss or gain in energy, the respective stoke or anti-stoke process, are the two possibilities resulting from Raman scattering. Stoke is the more intense of the two due to the natural existence of a greater number of ground state molecules compared to those in an excited state [74].

#### Raman spectrum and vibrations of chemical bonds

**Fig. 3:**
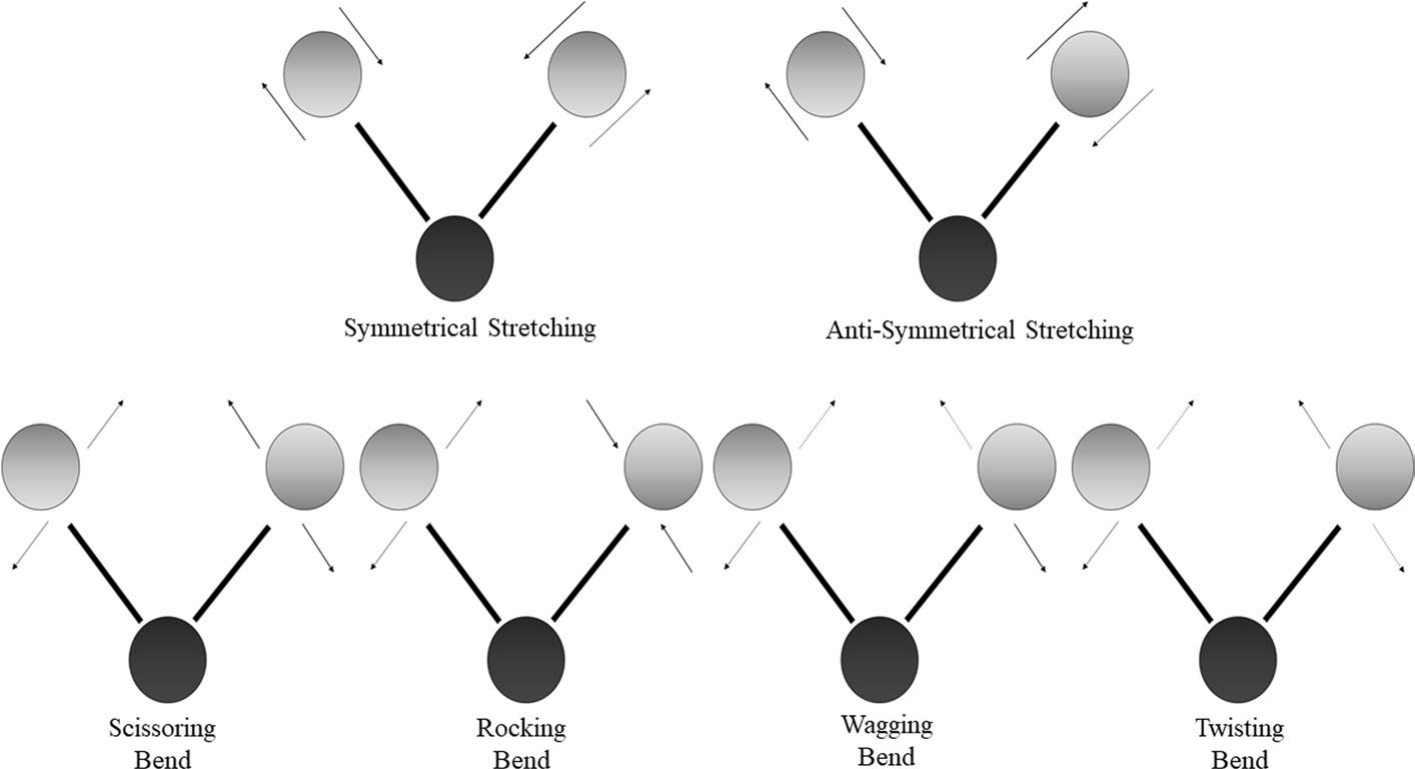
Classes of vibration

The Raman spectrum is a plot of the intensity of emitted photons against the Raman shift, determined as a shift in inverse wavelength and expressed with units of inverse length. The quantity, conventionally referred to as wavenumber, gives a direct link to energy. Chemical bonds offer specific vibrations and manifest as peaks in the Raman spectrum. The vibrations associated with chemical bonds can be divided into two groups: stretching and bending [75]. These two groups are further subdivided into more classes as depicted in Fig. 3. It is noticeable that these groups are bond length and angle dependent, where the bond length changes in stretching vibrations and angulations of the bands of wavelength become unstable in bending vibrations. The vibrations are correlated to specific molecular bonds, giving the biochemical fingerprints of the specimen. The symbolic representations of the respective types of vibrations are shown in Table 1.

**Table 1:** Symbols used to identify the sub-classes of vibrations

Symbol	Name of vibration
ν	Stretch
νs	Symmetric stretch
νas	Asymmetric stretch
δ	Deformation/bending/scissoring
ρ	Rock
τ	Torsion
ω	Wag
ωi	In-plane wag
ωo	Out-of-plane wag
t	Twist

#### Raman spectrometer

**Fig. 4:**
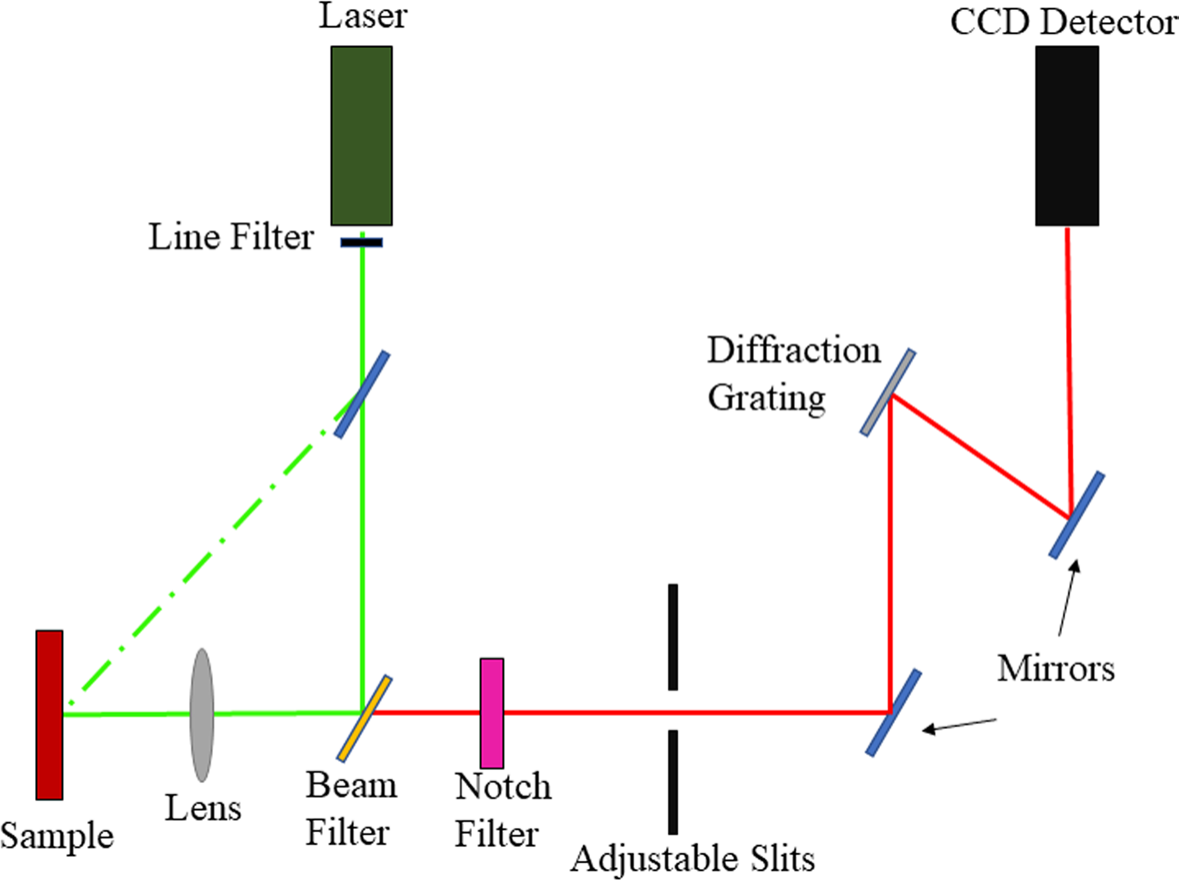
The basic components in a conventional Raman spectrometer

The construction of the Raman spectrometer relies on a monochromatic laser as the source of excitation. When supported by a light guidance system that incorporates lenses, mirrors and a diffraction grating to determine the pathway of the laser and scattered photons, this allows investigation of the sample with minimum power loss. Identification at the detector is in accordance with the wavelength. The presence of a notch filter is essential as it enhances the collection of Raman scattering and excludes the Rayleigh scattered photons emitted by the sample. A charge-coupled device (CCD) detector is typical, recording the signal and connecting to a computer system for data interpretation [76]. Figure 4 shows the schematic setup of a conventional Raman spectrometer. Various upgrades are available to better support the analysis of biological samples. For example, Raman microspectroscopy allows a conventional spectrometer to be embedded with a microscope to provide confocal capability [77]. Another advance is the Raman probe, an innovation allowing adaptation for in vivo studies [78].

### Raman spectroscopy in diagnosing bladder cancer

The implementation of Raman spectroscopy as an optical diagnostic tool for bladder cancer research offers various interesting opportunities. A major drawback of current cancer diagnostic tools is the inability to analyse the tumour at the cellular level. Most present-day techniques simply deliver information on the presence and location of the tumour. This inability is resolved by harnessing the power of Raman spectroscopy, providing minutiae of the molecular composition of the tissue and enabling the detection of biomolecular changes in the cells associated with tumour development. Accordingly, the remainder of this section seeks to give an outline summary of the determination of metabolic changes in bladder cancer cells, focusing on Raman spectroscopy studies conducted over the past decade.

#### Metabolite changes in bladder cancer

There is a clear trend in the determination of biomolecule changes in bladder cancer, with differentiation of healthy cells from cancer tissue, distinguishing high-grade bladder cancer tissue from low-grade cancer tissue. A summary of developments is provided in Additional file 1: Table S1, which consists of a list of observed Raman peaks with the corresponding assignments indicating the biomolecules and the vibrations of the functional group. The specific value of the intensity is not provided in the literature, and it is notable that the level of intensity is different for each study. However, the comparison of the intensity level, which is the indication of expression and concentration of metabolites, between the samples of healthy tissues, low-grade tumours and high-grade tumours enables us to differentiate the grading of bladder tumours.

There are various trends in terms of abnormalities of the metabolites found in cancer cells. It is evident that the concentrations of bio-substances, such as carbohydrates (C–C–C deformation), tryptophan (symmetric breathing), collagen, nucleic acid of DNA, protein/lipid (CH_2_ bending), fatty acid, amide I, triglyceride, and phenylalanine/tryptophan(C–C_6_H_5_) are greater in healthy tissue and lower in high-grade versus low-grade tumours. Furthermore, high-grade tumours have been reported to show overexpression of l-arginine, amide VI, l-tyrosine, phospholipid, d-mannos and α-helix protein compared to healthy tissue and low-grade tumours. Conversely, the metabolites phenylalanine (C–C vibration and twisting), DNA base, tyrosine, lipid (CH_3_, CH_2_ twisting) and the CH_3_, CH_2_ bonds of tryptophan, adenine and guanine show greater intensities in high-grade tumours, and are also decreased in normal tissue compared to low-grade tumours.

Moreover, an interesting pattern is identified wherein peaks not found in one group of tissue are detected at high intensity in other groups. For instance, for the Raman peaks of cholesterol and its esters, cytochromes and unsaturated fatty acids are more upregulated in low-grade tumours than in normal tissue, while they are absent in high-grade tumours. By contrast, olenic starch (C=C) appears in both low- and high-grade tumours but is not found in normal tissue. Accordingly, the establishment of Raman peaks as recognized in previous studies points to ways to differentiate normal bladder tissues from cancerous tissues and in classifying the grade of the tumour. Implementation of Raman spectroscopy is thus driving towards more detailed investigations of bladder cancer, observing the constitution of biocomponents at the molecular level.

#### Sensitivity and specificity of Raman spectroscopy in bladder cancer studies

Acceptance and employment of any diagnostic technique is strongly dependent upon the proffered sensitivity and specificity. In a medical context, sensitivity refers to the percentage of cases for which classification of a specific health condition is correct. Conversely, specificity refers to the percentage of cases producing correct negative signatures for a given health condition [85]. These two aspects are focal to successful diagnostic laboratory testing: the greater the percentages, the greater the efficiency of the device. Raman spectroscopy has been demonstrated to perform well. Table 2 provides a summary of the performance of Raman spectroscopy in categorizing healthy tissue versus low- and high-grade bladder cancer.

**Table 2:** Assessment of recent studies in the diagnosis bladder cancer (BC) in terms of the sensitivity and specificity provided by Raman spectroscopy

Type of sample	Raman spectrocopy instrumentation	Analysis technique	Healthy vs. bladder cancer		High-grade BC vs. low-grade BC		References
			Sensitivity (%)	Specificity (%)	Sensitivity (%)	Specificity (%)	
Tissue sample	Portable Raman spectrometer	PCA + LDA	85	79	–	–	[83]
Urine sample	Raman microscope	PCA	92	91	–	–	[84]
Tissue sample	Modulated Raman spectrometer	PCA	98	95	–	–	[86]
Blood serum	Surface enhanced Raman spectroscopy	GE + LDA	90.9	100		–	[87]
		PCA	74.6	97.2	–	–	
Blood serum	Surface enhanced Raman spectroscopy	SVM + RBF	–	–	92.3	98.2	[80]
Tissue sample	Raman microscope	PCA + kNN	–	–	99	87	[88]
Blood serum	Surface enhanced Raman spectroscopy	PLS + LDA	98.3	96.7	90.6	96.3	[81]

*PCA* principal component analysis, *LDA* linear discriminant analysis, *GE* generic algorithm, *SVM* support vector machine algorithm, *RBF* radial basis function analysis, *kNN* k nearest neighbor classification analysis

The assessments for studies made over the past decade were conducted with various samples, Raman spectroscopy instrumentations and analysis techniques. In discriminating bladder cancer from healthy tissue and supported by principal component and linear discriminant analysis (PCA and LDA), Draga et al. reported the in vivo use of a portable Raman spectroscopy system [83], obtaining respective sensitivity and specificity values of 85 of and 79%. Likewise supported by PCA, Shapiro et al. applied Raman molecular imaging technology in the investigation of urine samples, obtaining respective sensitivity and specificity values of 92 and 91% [84]. Canetta adopted the same analysis technique and introduced a modulated Raman spectrometer in differentiating normal from cancerous bladder tissues, with 98% sensitivity and 95% specificity [86]. Using surface-enhanced Raman spectroscopy (SERS), Li et al. investigated blood serum, adopting two types of analysis technique in classifying healthy versus cancerous bladder tissue [87]. In the use of PCA, respective sensitivity and specificity values of 74.6 and 97.2% were obtained. Using a generic algorithm (GE) combined with LDA, respective values of 90.9 and 100% were attained. Zhang et al. were able to identify high- and low-grade bladder tumours using blood serum and SERS [80]. They developed a classifier model from the support vector machine (SVM) algorithm, which allows spectral data classification and regression, combined with radial basis function (RBF), helped in yielding 92.3% sensitivity and 98.2% specificity. Bovenkamp et al. used a Raman microscope to examine bladder tissue, such that with the aid of a combination of PCA and k nearest neighbor classification analysis (kNN), high- and low-grade bladder tumours could be discriminated with 99 and 87% sensitivity and specificity, respectively [88]. Chen et al. successfully discerned normal bladder from cancerous tissue and high- from low-grade bladder cancer with respectivesensitivities and specificities of 98.3 and 96.7%, and 90.6 and 96.3% [81].

#### Independency of Raman spectroscopy on sample type

Additional file 1: Tables S1 and S3 clearly show that researchers verified the application of Raman spectroscopy on different biological sources in diagnosing bladder cancer. With respect to the specification mentioned, Kujdowicz et al. employed cultured cell lines of healthy bladder, low-grade and high-grade bladder cancer in their studies [79]. A portable Raman spectroscopy system is used by Draga et al. to perform the spectroscopy on the bladder lesion [83]. The achievement in discriminating the healthy bladder tissue, low-grade and high-grade bladder cancers from each other shows the aptitude of Raman spectroscopy in performing both in vitro and in vivo procedures. Several successful attempts can be found in conducting the Raman spectroscopy on samples that are discrete in types, such as tissue samples, urine samples and blood serum. Demonstrating high sensitivity and specificity and with no discernible dependence on sample type, Raman spectroscopy is seen as a promising diagnostic candidate for bladder cancer examination, with various analysis techniques and spectrometer setups enhancing the efficiency.

### Expected metabolites changes associated to FDFT1 in regulating chemoresistance

For cancer diagnosis via Raman spectroscopy, it is apparent that the biochemicals found in samples are rather large in range, varying with cancer tissue type and cancer cell and further involving the microenvironment of the cells and associations with the nucleus or cytoplasm. It should be noted that the samples are prepared via use of a cystoscope, embedded into microscope slides, and with FDFT1 staining performed by pathologists. In inspecting the chemoresistance exhibited by cancerous bladder tissue regulated by FDFT1, there are certain metabolites that are the focus of this study with Raman peak assignments for each.

In respect to the cholesterol biosynthesis pathway, the FDFT1 enzyme plays a key role in producing the squalene that leads to the opening up of the sterol branch leading to the eventual synthesis of cholesterol. Squalene, the direct product of FDFT1, can thus be expected in the FDFT1 samples extracted from the bladder. Furthermore, cholesterol is the end-product regulated by FDFT1, being found in almost all parts of the human body. It needs to be considered that the cholesterol regulated by FDFT1 has been found to influence the chemoresistance exerted by cancerous cells. Accordingly, the detection of trends in changes in cholesterol expression can be anticipated to lead to insights in understanding bladder cancer. In addition, activities occurring in the microenvironment of cancerous cells regulate the resistance towards cisplatin, where acceptance and rejection of the drug is mainly by the cell membrane. Hence, as it is the main biocompound of the phospholipid bilayer in exerting cell signaling and with association with cholesterol, unsaturated fatty acid is also predicted to be found in FDFT1 compounds.

The type of bonds present in the metabolites are listed in Additional file 1: Table S2 and the symbolic meanings connected to the Raman peak assignment vibrations are in Table 1. The Raman peak assignment for all three metabolites (squalene, cholesterol and unsaturated fatty acid) are listed in Additional file 1: Tables S3–S5 respectively, while Additional file 1: Figs. S1–S3 depict the chemical structures of the metabolites and the types of molecular bond that have been determined. The presence of other components in FDFT1 are to be anticipated, potentially leading to greater understanding of the chemoresistance exerted by bladder cancer.

### Supplementary Information

**Additional file 1: Table S1.** Raman peak assignments obtained from healthy bladder, low-grade bladder tumour and high-grade bladder tumour with symbols indicating, ‘/’: a peak with low intensity, ‘//’: a peak with greater intensity than ‘/’ but lower than ‘///’ and ‘///’: peak of high intensity. ‘X’ refers to the absence of the peak. **Table S2.** Molecular bonds denoted in Figs. S1 to S3. **Table S3.** Raman peak assignment for squalene. **Figure S1.** Chemical structure of squalene. **Table S4.** Raman peak assignment for cholesterol and its esters. **Figure S2.** Chemical structure of cholesterol. **Table S5.** Raman peak assignment for unsaturated fatty acid. **Figure S3.** Chemical structure of unsaturated fatty acids.

## Data Availability

All data analysed during this study are included in the published articles and books listed in the References list [1–95].

## Conclusion

Bladder cancer is a critical metastatic disease marked by a high rate of recurrence and poor overcall survival. To treat and control muscle invasive bladder cancers, cisplatin-based chemotherapy has become a favoured approach, offering the ability to alter the DNA of cancer cells. However, it has also been found that cancer cells can exhibit resistance towards chemotherapeutics, posing a threat to the effectiveness of cisplatin-based chemotherapy. Evidence reviewed herein indicates that biosynthesis of the intracellular cholesterol has the tendency inexerting resistance towards cancer treatments. Conversely, molecular studies have indicated that chemosensitivity in bladder cancer cells correlates with FDFT1 expression: the first key specific enzyme in intracellular cholesterol biosynthesis.

Using Raman Spectroscopy, this biochemical property could potentially be developed into a diagnostic or predictive tool to stratify patients with a high risk of developing resistance. Although cancer diagnostic technique have evolved, the inadequacy of contemporary diagnostic tools in inspecting the microenvironment of the cancerous cells can be overcome using Raman spectroscopy. This technique is proposed in current research as it provides information on biomolecular changes in tissue, detecting molecular bonds, often obtained with the high specificity and sensitivity that is vital for a diagnostic tool. The feasibility of this technique in analyzing biological specimens in solid, liquid, gases and gel forms indicates the lack of need for special sample preparation. Besides being accessible for both in vivo and in vitro measurements, enhancements in determining and differentiating the medical peculiarities from healthy conditions using Raman spectroscopy with the accommodation of signal processing techniques, has become a particularly strong advantage. Upregulated FDFT1 expression in cancer cells made changes in cholesterol biosynthesis and altered the cholesterol content of lipid rafts. Therefore, rapid multiplication and high survival rates occur in cancer cells. Thus, this project hypothesizes the Raman spectroscopy detection of high concentration of metabolites correlated to FDFT1 in chemoresistant bladder cancer tissue. The outcome will be significant biomarkers to differentiate chemoresistant from chemosensitive bladder tumours, which can be applied for further diagnosis and treatment procedures.

## Abbreviations

TURBT: Transurethral resection of bladder tumour
BC: Bladder cancer
NMIBC: Non-muscle invasive bladder cancer
MIBC: Muscle invasive bladder cancer
RC: Radical cystectomy
FDFT1: Farnesyl diphosphate farnesyltransferase 1
CT: Computed tomography
MRI: Modulated resonance imaging
LG: Low-grade
HG: High-grade
PUNLMP: Papillary urothelial neoplasm of low malignant potential
TNM: Tumour, node, metastasis
CIS: Carcinoma in situ
BCG: Bacillus Calmette Guerin
MVAC: Methotrexate, vinblastine, adriamycin, cisplatin
PFS: Progression-free survival
EMT: Epithelial–mesenchymal transition
LDL: Low-density lipoprotein
LDLR: Low-density lipoprotein receptor
ABC: ATP-binding cassette
CE: Cholesterol esters
ACAT: Acyl-coenzyme cholesterol acyltransferase
SREBP-2: Sterol regulatory element-binding protein-2
LXR: Liver X receptor
NRF1: Nuclear factor erythroid 2 related factor 1
ABCA: ATP-binding cassette subfamily A member 1
PTEN: Phosphatase and tensin homolog
FPP: Farnesyl diphosphate
CCD: Charge coupled device
PCA: Principal component analysis
LDA: Linear discriminant analysis
GE: Generic algorithm
SVM: Support vector machine
RBF: Radial basis function analysis
kNN: K nearest neighbour classification analysis
SERS: Surface enhanced Raman spectroscopy
