# Supplementary material for: Raman spectroscopy biochemical characterisation of bladder cancer cisplatin resistance regulated by FDFT1: a review
Source: Cell Mol Biol Lett. 2022 Jan 29;27:9. doi: 10.1186/s11658-022-00307-x (PMC8903573; doi:10.1186/s11658-022-00307-x)

**ADDITIONAL FILE 1**

**Raman Spectroscopy Biochemical Characterisation of Bladder Cancer Cisplatin Resistance Regulated by FDFT1: A Review**

Kanmalar, M^1^, Siti Fairus, AS^1^*, Nur Izzahtul Nabilla B Kamri^1^, Nur Akmarina BM Said^2^, Amirah Hajirah BA Jamil^2^, Kuppusamy, S^3^, Mun, KS^4^, Bradley, DA^5,6^

*^1^Department of Physics, Faculty of Science, University of Malaya, 50603 Kuala Lumpur, Malaysia*

*^2^Department of Pharmaceutical Life Sciences, Faculty of Pharmacy, University of Malaya, 50603 Kuala Lumpur, Malaysia*

*^3^Department of Surgery, University of Malaya, 50603 Kuala Lumpur, Malaysia*

*^4^Department of Pathology, Faculty of Medicine, University of Malaya, 50603 Kuala Lumpur, Malaysia*

*^5^Sunway University, Centre for Applied Physics and Radiation Technologies, Jalan University,*

*46150 PJ, Malaysia*

*^6^Department of Physics, University of Surrey, Guildford GU2 7XH, UK*

*Email:* [*s.fairus@um.edu.my*](mailto:s.fairus@um.edu.my)

**Table S1** Raman peak assignments obtained from healthy bladder, low-grade bladder tumour and high-grade bladder tumour with symbols indicating, ‘/’ : a peak with low intensity, ‘//’ : a peak with greater intensity than ‘/’ but lower than ‘///’ and ‘///’ : peak of high intensity. ‘X’ refers to the absence of the peak.

| **Raman shift (cm⁻¹)** | **Assignment/metabolites/chemical bond** | **Healthy bladder** | **Low-grade tumour** | **High-grade tumour** | **Reference** |
| --- | --- | --- | --- | --- | --- |
| 430 | Cholesterol and ester | X | X | / | [80] |
| 481 | C-C-C deformation of Carbohydrate | /// | // | / | [81] |
| 494 | L-Arginine | / | / | // | [82] |
| 579 | Carbohydrates | // | / | X | [80] |
| 589 | Amide VI | / | / | // | [82] |
| 621 | S-S Disulfide stretch, protein | / | // | /// | [83] |
| 639 | L-Tyrosine | / | / | // | [82] |
| 650 | C-C twisting mode of phenylalanine | / | // | /// | [81] |
| 680 | Ring breathing modes in DNA bases | / | // | /// | [84] |
| 683 | C-S twist of DNA (Guanine) | / | // | /// | [81] |
| 701 | Cholesterol and ester | / | // | X | [80] |
| 725 | C-H bending vibration mode of adenine | / | // | // | [82] |
| 751 | Cytochromes | / | // | X | [80] |
| 755 | Symmetric breathing, tryptophan | /// | // | / | [83] |
| 789 | DNA | / | // | /// | [84] |
| 875 | Phospholipid | / | / | // | [80] |
| 1025 | C-H stretch, phenylalanine | / | // | /// | [81] |
| 1067 | Phospholipid | / | / | // | [80] |
| 1080 | Collagen | /// | // | / | [84] |
| 1089 | Phospholipid | / | / | // | [80] |
| 1135 | D-mannos | / | / | // | [82] |
| 1170 | C-H in plane bending mode of tyrosine | / | // | /// | [84] |
| 1180 | Cytosine, guanine | / | // | /// |  |
| 1219 | C-C₆H₅, phenylalanine, tryptophan | /// | // | / | [81] |
| 1289 | Nucleic acid, DNA | /// | // | / | [84] |
| 1303 | CH₃, CH₂ twisting, collagen | /// | // | / |  |
| 1314 | CH₃, CH₂ twisting, lipid | / | // | /// | [81] |
| 1346 | CH₃CH₂ wagging of tryptophan, adenine, guanine | / | // | /// |  |
| 1370 | Saccharide band | / | // | /// | [84] |
| 1443 | Saturated fat | // | // | / | [82] |
| 1445 | CH₂ bending modes of proteins and lipids | /// | // | / | [81] |
| 1446 | CH₂ bending mode of protein, lipids | /// | // | / | [84] |
| 1560 | Tryptophan | / | // | /// |  |
| 1584 | C=C Phenylalanine/Olenic starch | X | / | / | [85] |
| 1588 | Cytochromes | / | // | X | [80] |
| 1610 | Cytosine (NH₂) | / | // | /// | [84] |
| 1654 | Amide I, α-helix protein | / | / | // | [82] |
| 1657 | Fatty acid, amide I, triglycerides | /// | // | / | [84] |
| 1660 | Unsaturated fatty acid | / | // | X | [80] |
| 1725 | Cholesterol and ester | X | X | / |  |
| 3015 | Unsaturated fatty acid | / | // | X |  |

**Table S2** Molecular bonds denoted in Figures S1 to S3.

| **Numbering in Figures S1 to S3** | **Molecular bond** |
| --- | --- |
| 1 | C-CH₃ |
| 2 | C=C-C |
| 3 | CH / =CH |
| 4 | C-C |
| 5 | CH₃ / =CH₃ |
| 6 | CH₂ / =CH₂ |
| 7 | C=C |
| 8 | C-O-O |
| 9 | Cholesterol ring |
| 10 | CH₂ in ring |

**Table S3** Raman peak assignment for squalene

| **Raman shift (cm⁻¹)** | **Vibration assignment** | **Reference** |
| --- | --- | --- |
| 454 | ωo (C-CH₃), δ (C=C-C) |  |
| 804 | ωo (CH), ν (C-C) |  |
| 1003 | ρ (CH₃), ωo (CH) |  |
| 1281 | δ (CH₃), t (CH₂) | [93] |
| 1330 | δ (CH₃), ω (CH₂), ωi (CH) |  |
| 1382 | δ (CH₃), ωi (CH), v(C-C) |  |
| 1451 | δ (CH₃), δ (CH₂) |  |
| 1668 | ν (C=C) |  |
| 1670 | νs (C=C) | [94] |
| 2913 | ν (CH₃), ν (CH₂) | [93] |
| 2916 | ν (C-H) | [94] |

**Figure S1** Chemical structure of squalene


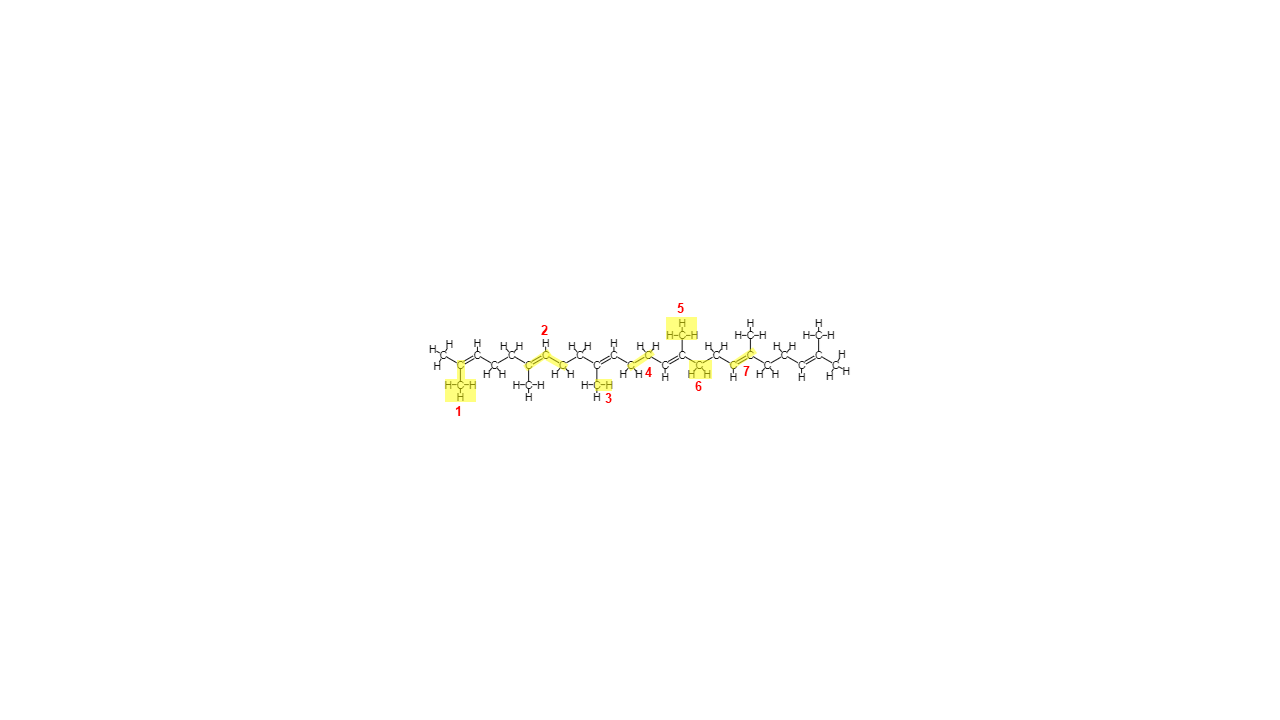


**Table S4** Raman peak assignment for cholesterol and its esters

| **Raman shift (cm⁻¹)** | **Vibration assignment** | **Reference** |
| --- | --- | --- |
| 424 | Cholesterol β(CH₂) in ring |  |
| 429 | Cholesteryl oleate β (CH₂) in ring |  |
| 430 | Cholesteryl stearate β (CH₂) in ring |  |
| 431 | Cholesteryl linoleate β (CH₂) in ring |  |
| 548 | Cholesterol β (CH₂) in ring | [92] |
| 701 | Cholesterol, cholesterol ring deformation |  |
| 702 | Cholesteryl palmitate, cholesterol ring deformation |  |
| 702 | Cholesteryl stearate, cholesterol ring deformation |  |
| 703 | Cholesteryl linoleate, cholesterol ring deformation |  |
| 703 | Cholesterol, sterol ring stretch | [93] |
| 704 | Cholesteryl oleate, cholesterol ring deformation | [92] |
| 746 | Cholesterol, sterol ring stretch |  |
| 759 | Cholesterol, sterol ring stretch |  |
| 881 | Cholesterol, sterol ring stretch | [93] |
| 962 | Cholesterol, sterol ring stretch |  |
| 1066 | Cholesteryl palmitate ν (C-C) |  |
| 1068 | Cholesteryl stearate ν (C-C) |  |
| 1087 | Cholesterol ν (C-C) | [92] |
| 1130 | Cholesterol ν (C-C) |  |
| 1131 | Cholesteryl palmitate ν (C-C) |  |
| 1132 | Cholesterol ν (C-C) | [93] |
| 1133 | Cholesteryl stearate ν (C-C) | [92] |
| 1178 | Cholesterol ν (C-C) |  |
| 1179 | Cholesterol ν (C-C) | [93] |
| 1261 | Cholesteryl oleate δ (=CH) |  |
| 1263 | Cholesteryl linoleate δ (=CH) | [92] |
| 1298 | Cholesteryl palmitate τ (CH₂) |  |
| 1300 | Cholesterol, δ (CH₂) and δ (=CH) | [93] |
| 1301 | Cholesteryl stearate τ (CH₂) |  |
| 1306 | Cholesteryl Ooeate τ (CH₂) |  |
| 1306 | Cholesteryl linoleate τ (CH₂) | [92] |
| 1428 | Cholesteryl stearate β (CH₂) |  |
| 1437 | Cholesterol δ (C-H) | [94] |
| 1440 | Cholesterol δ (CH₂), δ (CH₃) | [95] |
| 1441 | Cholesteryl oleate α (CH₂/CH₃) |  |
| 1442 | Cholesterol α (CH₂/CH₃) |  |
| 1442 | Cholesteryl palmitate α (CH₂/CH₃) |  |
| 1442 | Cholesteryl linoleate α (CH₂/CH₃) |  |
| 1444 | Cholesteryl stearate α (CH₂/CH₃) | [92] |
| 1464 | Cholesteryl palmitate β (CH₂/CH₃) |  |
| 1468 | Cholesteryl stearate β (CH₂/CH₃) |  |
| 1659 | Cholesteryl oleate ν (C = C) |  |
| 1662 | Cholesteryl linoleate ν (C = C) |  |
| 1667 | Cholesterol ν (C=C) | [94] |
| 1668 | Cholesteryl palmitate ν (C = C) | [92] |
| 1670 | Cholesteryl oleate ν (C = C) |  |
| 1670 | Cholesterol ν (C=C) | [95] |
| 1671 | Cholesteryl stearate ν (C = C) |  |
| 1672 | Cholesterol ν (C = C) |  |
| 1739 | Cholesteryl palmitate ν (C = O) |  |
| 1739 | Cholesteryl oleate ν (C = O) |  |
| 1741 | Cholesteryl stearate ν (C = O) |  |
| 1742 | Cholesteryl linoleate ν (C = O) |  |
| 2846 | Cholesteryl palmitate νs (=CH₂) |  |
| 2846 | Cholesteryl oleate νs (=CH₂) |  |
| 2852 | Cholesteryl stearate νs (=CH₂) |  |
| 2864 | Cholesterol νs (=CH₂) | [92] |
| 2865 | Cholesteryl linoleate νs (=CH₂) |  |
| 2866 | Cholesteryl oleate νs (=CH₂) |  |
| 2881 | Cholesteryl Palmitate νas (=CH₂) |  |
| 2885 | Cholesteryl stearate νas (=CH₂) |  |
| 2930 | Cholesterol νs (=CH₃) |  |
| 2937 | Cholesteryl palmitate νs (=CH₃) |  |
| 2937 | Cholesteryl linoleate νs (=CH₃) |  |
| 2957 | Cholesteryl palmitate νas (=CH₃) |  |
| 3009 | Cholesteryl oleate ν (=CH) |  |
| 3013 | Cholesteryl linoleate ν (=CH) |  |

**Figure S2** Chemical structure of cholesterol


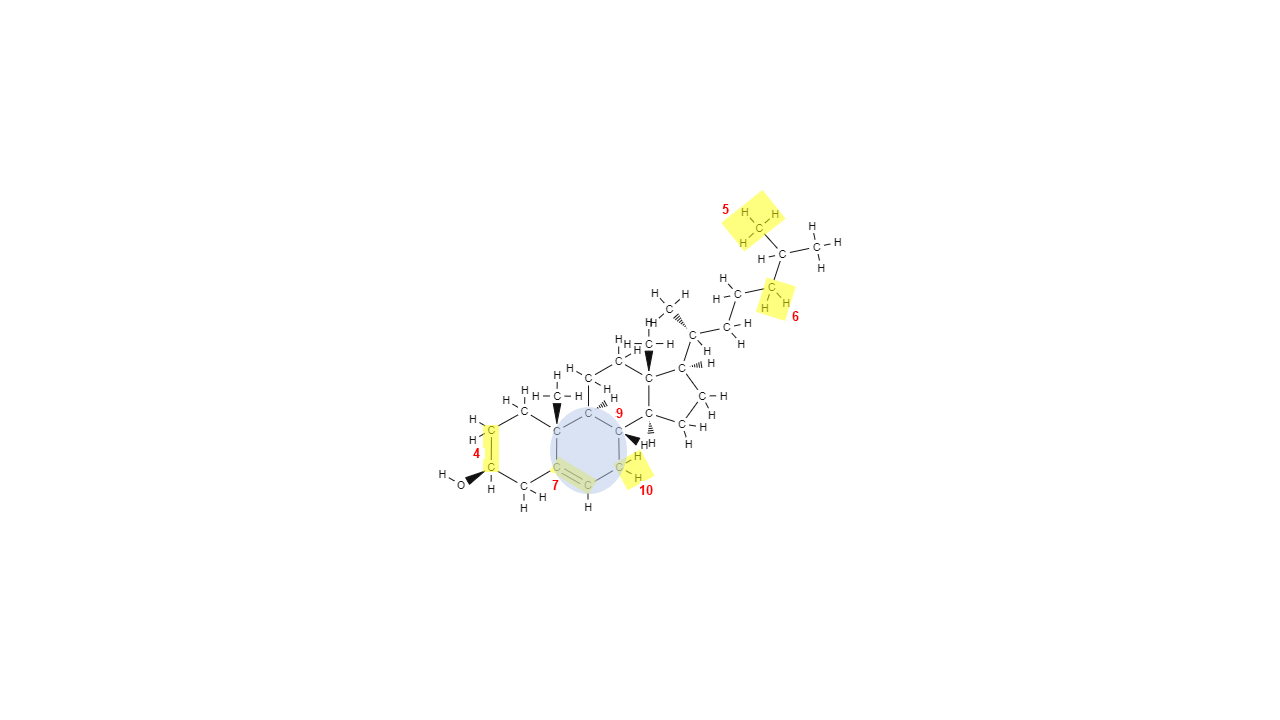


**Table S5** Raman peak assignment for unsaturated fatty acid

| **Raman shift (cm⁻¹)** | **Vibration assignment** | **Reference** |
| --- | --- | --- |
| 867 | α-linolenic acid (C-O-O) skeleton |  |
| 872 | Linoleic acid (C-O-O) skeleton |  |
| 894 | Palmitoleic acid (C-O-O) skeleton |  |
| 904 | Linoleic acid β (CH) |  |
| 909 | Elaidic acid β (CH) |  |
| 935 | Arachidonic acid β (CH) |  |
| 971 | α-Linolenic acid β (CH) | [92] |
| 972 | Linoleic acid β (CH) |  |
| 974 | Arachidonic acid β (CH) |  |
| 975 | Palmitoleic acid β (CH) |  |
| 976 | Oleic acid β (CH) |  |
| 1064 | Arachidonic acid ν (C-C) |  |
| 1065 | Elaidic acid ν (C-C) |  |
| 1068 | α-Linolenic acid ν (C-C) |  |
| 1075 | Linoleic acid ν (C-C) |  |
| 1084 | Oleic acid ν (C-C) |  |
| 1086 | Palmitoleic acid ν (C-C) |  |
| 1086 | α-Linolenic acid ν (C-C) |  |
| 1087 | Linoleic acid ν (C-C) |  |
| 1098 | Elaidic acid ν (C-C) |  |
| 1107 | Linoleic acid ν (C-C) |  |
| 1111 | Arachidonic acid ν (C-C) | [92] |
| 1118 | Palmitoleic acid ν (C-C) |  |
| 1120 | Oleic acid ν (C-C) |  |
| 1124 | Elaidic acid ν (C-C) |  |
| 1159 | Arachidonic acid ν (C-C) |  |
| 1166 | Elaidic acid ν (C-C) |  |
| 1260 | α-Linolenic acid δ (=CH) |  |
| 1260 | Arachidonic acid δ (=CH) |  |
| 1262 | Linoleic acid δ (=CH) |  |
| 1262 | δ (=C-H) | [96] |
| 1265 | Palmitoleic acid δ (=CH) |  |
| 1265 | Oleic acid δ (=CH) |  |
| 1287 | Elaidic acid δ (=CH) |  |
| 1296 | Arachidonic acid τ (CH₂) |  |
| 1300 | Linoleic acid τ (CH₂) | [92] |
| 1300 | α-Linolenic acid τ (CH₂) |  |
| 1303 | Elaidic acid τ (CH₂) |  |
| 1305 | Palmitoleic acid τ (CH₂) |  |
| 1306 | Oleic acid τ (CH₂) |  |
| 1420 | Elaidic acid β (CH₂) |  |
| 1437 | α-Linolenic acid α (CH₂/CH₃) |  |
| 1438 | Linoleic acid α (CH₂/CH₃) |  |
| 1438 | Arachidonic acid α (CH₂/CH₃) |  |
| 1438 | t (CH₂) | [96] |
| 1441 | Elaidic acid α (CH₂/CH₃) |  |
| 1444 | Palmitoleic acid α (CH₂/CH₃) |  |
| 1444 | Oleic acid α (CH₂/CH₃) |  |
| 1464 | Elaidic acid β (CH₂/CH₃) | [92] |
| 1653 | Arachidonic acid ν (C = C) |  |
| 1654 | Linoleic acid ν (C = C) |  |
| 1654 | α-Linolenic acid ν (C = C) |  |
| 1655 | Palmitoleic acid ν (C = C) |  |
| 1655 | ν (C=C) | [96] |
| 1657 | Oleic acid ν (C = C) | [92] |
| 1668 | Elaidic acid ν (C=C) | [96] |
| 1672 | Elaidic acid ν (C = C) |  |
| 2844 | Elaidic acid νs (=CH₂) |  |
| 2845 | Linoleic acid νs (=CH₂) | [92] |
| 2848 | α-Linolenic acid νs (=CH₂) |  |
| 2849 | Palmitoleic acid νs (=CH₂) |  |
| 2850 | ν (C-H) in CH₂ | [96] |
| 2852 | Oleic acid νs (=CH₂) |  |
| 2862 | Arachidonic acid νs (=CH₂) |  |
| 2880 | Elaidic acid νas (=CH₂) |  |
| 2885 | Linoleic acid νas (=CH₂) |  |
| 2886 | Arachidonic acid νas (=CH₂) |  |
| 2888 | α-Linolenic acid νas (=CH₂) |  |
| 2891 | Oleic acid νas (=CH₂) |  |
| 2895 | Palmitoleic acid νas (=CH₂) |  |
| 2920 | Oleic acid νs (=CH₃) |  |
| 2921 | Elaidic acid νs (=CH₃) | [92] |
| 2923 | α-Linolenic acid νs (=CH₃) |  |
| 2923 | Arachidonic acid νs (=CH₃) |  |
| 2924 | Palmitoleic acid νs (=CH₃) |  |
| 2929 | Linoleic acid νs (=CH₃) |  |
| 2935 | ν (C-H) in CH₃ | [96] |
| 3002 | Linoleic acid ν (=CH) |  |
| 3002 | α-Linolenic acid ν (=CH) |  |
| 3003 | Arachidonic acid ν (=CH) | [92] |
| 3004 | Oleic acid ν (=CH) |  |
| 3005 | Palmitoleic acid ν (=CH) |  |
| 3005 | Cholesterol ν (=C-H) | [96] |

**Figure S3** Chemical structure of unsaturated fatty acids


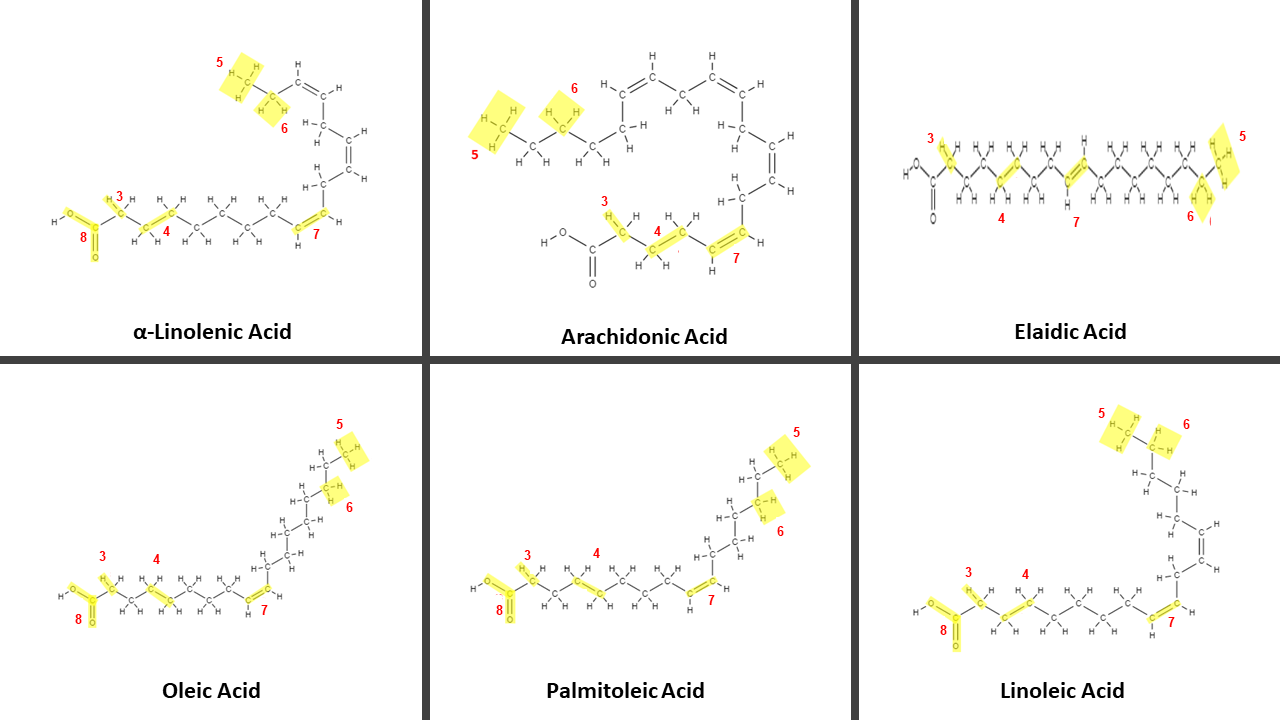

Supplement: Supplementary file 1 — Additional file 1: Table S1. Raman peak assignments obtained from healthy bladder, low-grade bladder tumour and high-grade bladder tumour with symbols indicating, ‘/’: a peak with low intensity, ‘//’: a peak with greater intensity than ‘/’ but lower than ‘///’ and ‘///’: peak of high intensity. ‘X’ refers to the absence of the peak. Table S2. Molecular bonds denoted in Figs. S1 to S3. Table S3. Raman peak assignment for squalene. Figure S1. Chemical structure of squalene. Table S4. Raman peak assignment for cholesterol and its esters. Figure S2. Chemical structure of cholesterol. Table S5. Raman peak assignment for unsaturated fatty acid. Figure S3. Chemical structure of unsaturated fatty acids. [file 11658_2022_307_MOESM1_ESM.docx]
